# Characterization of Conserved Evolution in H7N9 Avian Influenza Virus Prior Mass Vaccination

**DOI:** 10.1080/21505594.2024.2395837

**Published:** 2024-09-06

**Authors:** Dongchang He, Xiyue Wang, Huiguang Wu, Kairui Cai, Xiaoli Song, Xiaoquan Wang, Jiao Hu, Shunlin Hu, Xiaowen Liu, Chan Ding, Daxin Peng, Shuo Su, Min Gu, Xiufan Liu

**Affiliations:** a Animal Infectious Disease Laboratory, College of Veterinary Medicine, Yangzhou University, Yangzhou, Jiangsu, China; b College of Veterinary Medicine, Jiangsu Agri-animal Husbandry Vocational College, Taizhou, China; c Animal Epidemic Prevention Office, Jiangsu Provincial Animal Disease Control Center, Nanjing, Jiangsu, China; d Jiangsu Co-innovation Center for Prevention and Control of Important Animal Infectious Diseases and Zoonosis, Yangzhou University, Yangzhou, China; e Jiangsu Key Laboratory of Zoonosis, Yangzhou University, Yangzhou, China; f Department of Avian Diseases, Shanghai Veterinary Research Institute, Chinese Academy of Agricultural Sciences, Shanghai, China; g College of Veterinary Medicine, Nanjing Agricultural University, Nanjing, China

**Keywords:** Avian influenza, H7N9, evolution, vaccination, elimination

## Abstract

Vaccination is crucial for the prevention and mitigation of avian influenza infections in China. The inactivated H7N9 vaccine, when administered to poultry, significantly lowers the risk of infection among both poultry and humans, while also markedly decreasing the prevalence of H7N9 detections. Highly pathogenic (HP) H7N9 viruses occasionally appear, whereas their low pathogenicity (LP) counterparts have been scarcely detected since 2018. However, these contributing factors remain poorly understood. We conducted an exploratory investigation of the mechanics via the application of comprehensive bioinformatic approaches. We delineated the Yangtze River Delta (YRD) H7N9 lineage into 5 clades (YRD-A to E). Our findings highlight the emergence and peak occurrence of the LP H7N9-containing YRD-E clade during the 5th epidemic wave in China’s primary poultry farming areas. A more effective control of LP H7N9 through vaccination was observed compared to that of its HP H7N9 counterpart. YRD-E exhibited a tardy evolutionary trajectory, denoted by the conservation of its genetic and antigenic variation. Our analysis of YRD-E revealed only minimal amino acid substitutions along its phylogenetic tree and a few selective sweep mutations since 2016. In terms of epidemic fitness, the YRD-E was measured to be lower than that of the HP variants. Collectively, these findings underscore the conserved evolutionary patterns distinguishing the YRD-E. Given the conservation presented in its evolutionary patterns, the YRD-E LP H7N9 is hypothesized to be associated with a reduction following the mass vaccination in a relatively short period owing to its lower probability of antigenic variation that might affect vaccine efficiency.

## Introduction

Since the zoonotic H7N9 avian influenza virus (AIV) was first detected in poultry in 2013 in the Yangtze River Delta (YRD), it has caused significant public health and economic concerns The virus has been responsible for at least five epidemic waves, resulting in 616 deaths out of 1568 human infections [1]. Shortly after its emergence, H7N9 spread to the Pearl River Delta (PRD) region, leading to the identification of two primary sources of H7N9 outbreaks in these regions. The continuous evolution of the virus has led to the establishment of two genetically distinct lineages: the YRD and PRD lineages [2, 3].

In the middle of 2016 (wave 4), the low pathogenic (LP) H7N9 virus from the YRD lineage mutated, acquiring a 4 amino acid insertion in the hemagglutinin (HA) protein and became highly pathogenic (HP) in the PRD region (Guangdong). Low pathogenic (LP) avian influenza viruses typically cause mild symptoms in poultry, while highly pathogenic (HP) strains can cause severe disease and high mortality rates [4–6]. According to a previous study, five YRD (A-E) and two PRD (A-B) clusters were identified according to the HA phylogeny as of 2019 [7]. The YRD-C HP H7N9 and YRD-E LP H7N9 viruses played significant roles in the largest H7N9 outbreak during 2016–2017 (wave 5).

In response to the sharp increase in human infections and economic losses in the poultry industry during the 2017 epidemic, a vaccination campaign was launched using a recombinant H5/H7 bivalent inactive vaccine (H5N1 Re-8 and H7N9 Re-1). The vaccination campaign, first introduced in Guangxi and Guangdong in July 2017 (http://www.moa.gov.cn/xw/qg/201708/t20170807_5770956.htm), and expanded to the rest of mainland China in September 2017, aimed to control the spread of the virus [8]. According to the administrative guidelines published by the Chinese Ministry of Agriculture and Rural Affairs (http://www.moa.gov.cn/nybgb/2018/201803/201805/t201805286143196.htm), more than 90% poultry vaccination coverage and HI titers ≥ 4 against H7N9 are required. This national vaccination program has effectively controlled outbreaks and circulation of H7N9 viruses in both poultry and humans [5, 9–11].

The most recent LP H7N9 AIV caused human infections in January 2018 (A/Xinjiang/04062/2018). The last isolation of LP H7N9 AIV in chickens occurred in November 2017 (A/chicken/Hunan/S31330/2017) [7]. Since then, LP H7N9 viruses have been rarely detected in poultry farms or live bird markets (LBMs), suggesting that the LP H7N9 AIV may have disappeared due to the intensive vaccination efforts in China. However, HP H7N9 AIVs have continued to circulate and evade the vaccination program in poultry farms since 2018 [1,12–16]. The mechanisms behind the disappearance of LP and the persistence of HP H7N9 AIVs remain unclear.

The surface proteins of the influenza virus, particularly the HA protein, continually evolve and adapt when circulating in natural hosts. The HA protein is the main target of neutralizing antibodies and mediates binding to host cell receptors. Selection pressures, such as receptor-binding avidity [17] and evasion of humoral immunity [18], drive the fixation of mutations in HA. Therefore, to understand the adaptive evolution of H7N9 AIVs and the potential reasons for the disappearance of LP H7N9 AIV, we analyzed the HA genes of H7N9 isolates collected between 2013 and 2019 using datasets from our previous study [7,19]. In this study, we evaluate the H7N9 population structure, antigenic variation estimated through sequence analysis, adaptive mutations, population fitness, and *dN/dS* accumulation ratios of H7N9 AIVs.

## Methods

### H7N9 spatiotemporal distribution in wave 5

H7N9 nomenclature clades and datasets downsampled by CD-HIT (v4.8.1) with thresholds of 99.9% (cd999 dataset) or 99% (cd99 dataset) were obtained from our previous studies [7,19]. The dataset spans from 2013 to 2019, encompassing sequences from the Influenza Virus Database and the Global Initiative on Sharing All Influenza Data (GISAID). A Sankey plot was prepared to depict the waves, clades, and hosts of the H7N9 viruses. To analyze the viral spread of the YRD-E clade in Wave 5 (October 2016 to September 2017), we extracted the isolation date and location (province and municipality) from the dataset and plotted them on a map. Wave 5 has been divided into 4 periods: Period 1 (2016.10–2016.12), Period 2 (2017.1–2017.3), Period 3 (2017.4–2017.6), and Period 4 (2017.7–2017.9). Weekly accumulation charts of H7N9 isolates and antibody titers before and after vaccination were pooled from 2013 to 2019 and plotted.

### Antibody titers of H7N9 vaccine in 2017

Data on individual antibody titers were collected from different bird species at different sites in Jiangsu Province before and after the start of vaccination (data source: Jiangsu Provincial Animal Disease Control Center). The individual antibody hemagglutination inhibition titer ≥4 was considered positive. Sampling sites included breeding farms, commercial poultry farms, backyard poultry, live bird markets, and other places (harmless treatment centers, environment). Most samples were collected from chicken, duck, and goose species, with additional samples from pigeons, quails, mandarin ducks, and wild birds. All figures were plotted using ggplot2 [20], tidyverse [21], etc. in R (v4.1.0).

### Evolutionary rate

To assess the evolution of H7N9 viruses, we performed root-to-tip regression with TreeTime (v0.10, https://treetime.biozentrum.unibas.ch) [22] to determine the temporal signal. Subsequently, the evolutionary rates for the Pearl River Delta (PRD), YRD-C, and YRD-E clades were evaluated independently using BEAST(v1.10.4, https://github.com/beast-dev/beast-mcmc) [23], selecting the General Time Reversible (GTR+G4) model as recommended by ModelFinder in Phylosuite (v1.2.3, https://github.com/dongzhang0725/PhyloSuite) [24] to accurately reflect the substitution patterns observed in H7N9 sequences. The strict molecular clock model was appropriate due to the relatively brief evolutionary intervals in our study, ensuring uniform rate estimates across the phylogenetic tree. The Bayesian SkyGrid tree prior [23], selected for its capacity to flexibly represent demographic shifts, is particularly useful for tracking the virus population changes prompted by external interventions like vaccination. The MCMC chain lengths were set to 200 million generations, with trees collected every 20,000 steps in BEAST. Multiple BEAST runs were performed, using the same estimates. The log files obtained from the same XML were merged using LogCombiner (v1.10.4) and examined using Tracer (v1.7.1) to reach convergence (Effective Sample Size >200).

### Discriminant analysis of principal components

To depict the genetic clusters of the YRD lineage, we used Discriminant Analysis of Principal Components (DAPC) to infer viral population structure [25]. Data were first transformed using principal component analysis (PCA), and clusters were identified using discriminant analysis (DA). Viruses were divided and colored by outbreak waves (waves 1–7) in ellipses. The analysis code was adapted from the adegenet tutorial (https://grunwaldlab.github.io/Population_Genetics_in_R/DAPC.html) [25,26] and executed in RStudio.

### Hamming distance of antigenic sites

Although the cross-hemagglutination inhibition test is the gold standard for testing for influenza antigenic variation, this technique is cost- and time-intensive, making it impossible to identify all strains [27]. Therefore, we used the Hamming distance based on the amino acid sequence of the antigenic sites to evaluate the antigenicity variation in the YRD lineage. Antigenic sites of the H7N9 HA gene were referenced from published literature [28], including Antigenic site A (full-length HA numbering): 130, 132, 134, 138–146, 148–153, 157, 159, 177; Antigenic site B: 136, 137, 162–165, 168, 169, 172–174, 195–199, 201, 203, 205–207; Antigenic site C: 52–56, 58–59, 61–62, 282, 284, 285, 287–289, 303, 306, 308, 309, 313, 314, 316–321; Antigenic site D: 104, 110–111, 125, 129, 176, 179–186, 188, 191, 210, 216–218, 220–228, 235–239, 247, 249, 251, 253, 255–257; and Antigenic site E: 65, 67, 70, 71, 75, 83, 86, 88–91, 94–96, 99, 100, 117, 269–270, 271, 274. The Hamming distance was calculated based on the reference strain A/Shanghai/1/2013 (SH1). The analytical code was obtained from Dr. Christopher S. Anderson [29] and modified for this study. Scatter plots were plotted using ggplot2, with the horizontal axis representing time and the vertical axis representing cumulative Hamming distance values. A smoothed regression fitted curve was added to the scatter plots using a generalized additive model (GAM).

### Ancestral sequence reconstruction

To investigate amino acid substitutions along the branches of the HA phylogenetic tree, we used Treesub (https://github.com/tamuri/treesub) to reconstruct ancestral sequences and analyze nonsynonymous mutations based on a previous study [1]. The annotation tree generated by Treesub was read using an in-house code with Treeio [30] and visualized using ggtree [31,32].

### Sweep dynamics

The directed evolution process promotes increased fitness of individuals through the introduction of favorable amino acid mutations, and the corresponding alleles increase in frequency in viral populations, leading to a decrease in diversity, a process known as selective sweep [33]. The Sweep Dynamics (SD) plot is a computational method that employs phylogenetic algorithms and statistical techniques to evaluate the molecular adaptability of rapidly evolving viruses using longitudinal sequence data [34]. The SD plot helps identify selective sweeps in certain periods and associated mutations that can provide adaptive advantages to viruses. We utilized the HA sequence dataset (*n* = 1053) of the YRD lineage LP H7N9 strains to analyze the selective sweep using SDplots in Docker, with SH1 as the reference sequence. Mutations that significantly increased the frequency of isolations over consecutive months were identified as possible sites that could provide a selective advantage for the viral population.

### Local branching index

The local branching index (LBI) was used to analyze the shape and branching pattern of the tree to assess the epidemic fitness of the viruses, providing insights into their evolutionary dynamics [35,36]. LBI was normalized to 0–1, with 0 representing poor fitness and 1 representing good fitness. To investigate the fitness of individual H7N9 strains, an HA evolutionary tree of cd99 data was constructed using NextStrain (https://nextstrain.org/) [37]. A preliminary tree was constructed using Augur (v12.0.0) and IQ-Tree [38,39]. A time-resolved tree and ancestral sequence reconstruction were performed in a joint inference model using Treetime (v0.8.5) [22]. To calculate the LBI for each strain, the time-scale parameter τ (tau) for local tree length estimation was set to 0.3. The results were visualized using the Auspice [37,39].

### Quantification of nonsynonymous to synonymous divergence ratio

To determine the divergence ratio, we estimated the accumulation of nonsynonymous to synonymous (*dN/dS*) ratios in several clades. Hamming distance divergences were determined between the reference sequence (A/Shanghai/02/2013) and differences between the HA clades (PRD-A, PRD-B, YRD-C, and YRD-E) from March 2013 to October 2019 in non-overlapping six-month time windows. The code was adapted from Kistler et al.. (https://github.com/blab/sarscov2-adaptive-evolution/blob/master/analysis/fig2-divergence.ipynb) and modified for this study.

## Results

### LP H7N9 predominated in China’s main poultry-rearing area and peaked in wave 5

**Figure 1.**
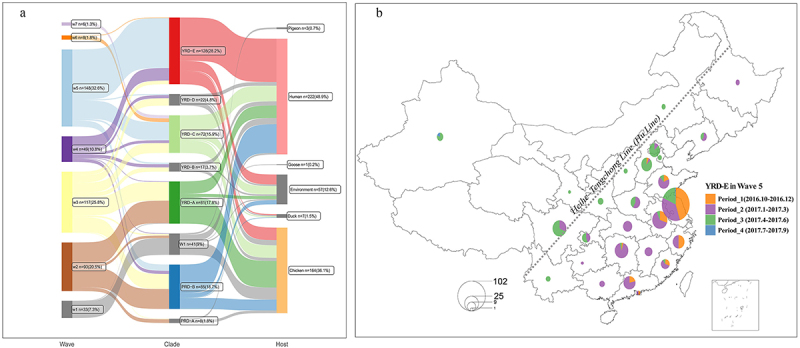
Sankey plot of H7N9 and spatiotemporal distribution of the YRD-E clade. (a) The proportions of the outbreak waves, clades, and hosts are shown in a Sankey plot. (b) The number of YRD-E clade isolates and epidemic range in the largest outbreak wave 5 are shown on the map. The periods and viral isolates in each province are depicted in pie charts.

By analyzing the metadata (outbreak waves, clades, and hosts) of H7N9 viruses, we found that the YRD-E clade LP H7N9 viruses, which emerged in wave 3 and peaked in the largest outbreak wave 5 (Figure 1a), were predominant. The predominance of LP H7N9 was determined based on the frequency of detection in poultry and human samples, as well as the absence of significant outbreaks post-vaccination. More than 64% of the strains in the YRD-E clade were isolated in wave 5. Moreover, a considerable proportion of viruses in the YRD-E and PRD-B clades cause human infections. According to the results of the spatiotemporal distribution analysis (Figure 1b), the YRD-E clade has mostly been found in the eastern area of China’s Heihe – Tengchong line, which has a high poultry population density [40]. The YRD-E clade peaked in South and Central China during Period 2 of wave 5 (January–March 2017). The number of YRD-E viruses significantly decreased in South and Central China, while it noticeably increased in North China after March 2017 (Period 3: April – June 2017). The decay of the H7N9 isolates in the south declined earlier than that in North China. Subsequently, the number of isolates and the virus population size unexpectedly decreased nationwide after July 2017 (Period 4). Our results showed that the YRD-E clade LP H7N9 was predominant in wave 5 in the main poultry-rearing area.

### Vaccination wipes out LP rather than HP H7N9

**Figure 2.**
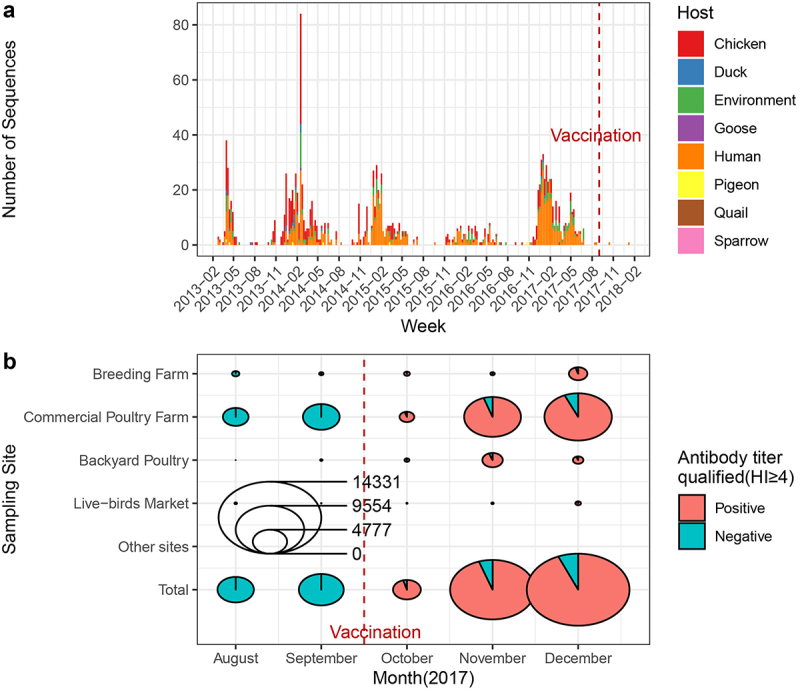
Stacked bar chart of H7N9 sequence number against onset week in China, and the pie charts of variant bird species vaccination associated qualified antibody titer (HI ≥ 4) in Jiangsu Province around September 2017(Data source: Jiangsu provincial animal disease control center). (a) The x-axis shows the week of onset among all hosts infected with the H7N9 virus (*n* = 1514). Hosts are shown in different colors and stacked on the y-axis. (b) Antibody titer qualification proportion was depicted in a pie chart. The sampling sites included breeding farms, commercial poultry farms, backyard poultry, live-bird markets, and other sites (e.g. harmless treatment centers). environment). Samples were primarily collected from chicken, duck, and geese. Other bird species (e.g. pigeons, quails, mandarin ducks, and wild birds) may also be involved. The red dashed line indicates the timepoint of the H7N9 Re-1 vaccine’s initialization.

Metadata analysis revealed that H7N9 has caused more than five epidemic waves since its emergence in 2013, resulting in numerous poultry and human infections (Figure 2a). H7N9-related antibodies were almost absent before vaccination based on the collected surveillance data (Figure 2b). After the nationwide introduction of the H7N9 Re-1 vaccine in poultry in September 2017, the individual antibody positivity rates (hemagglutination inhibition titer ≥4) in the sampling of H7N9-associated antibody tests were 95.76% (October), 94.81% (November), and 93.71% (December) (Figure 2b). These antibody positivity rates were higher than the 70% rate required by the National Administrative Guidance (http://www.moa.gov.cn/nybgb/2018/201803/201805/t201805286143196.htm). Therefore, based on a parsimonious explanation, we believe that the H7N9 vaccine has contributed to the eradication of the LP H7N9 virus in poultry at least regionally. Following the introduction of mass vaccination, a notable decrease in the detection of LP H7N9 was observed (Figure 2a), suggesting a potential association between vaccination efforts and virus prevalence. Gradually, LP pathogenic H7N9 in poultry was rarely isolated or detected nationwide after 2018. However, highly pathogenic H7N9 viruses persist and circulate primarily into poultry.

### Slow evolution trajectory of the YRD-E LP H7N9

**Figure 3.**
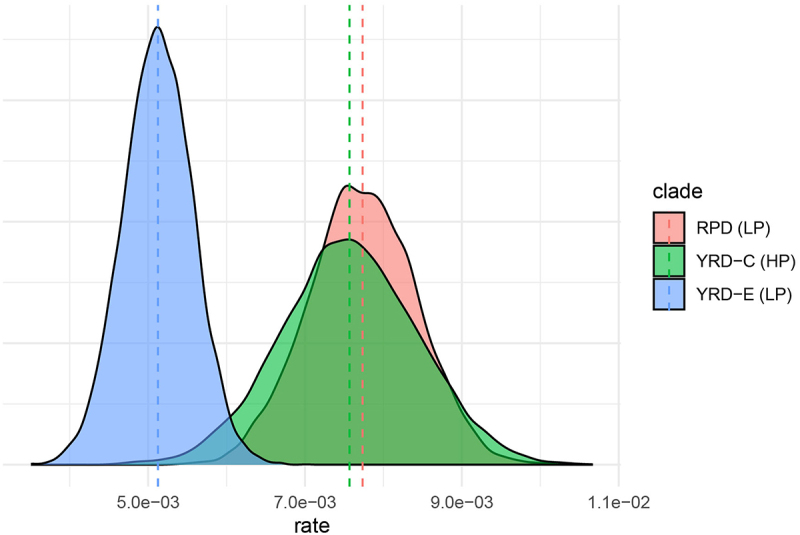
Evolution rate of the H7N9 variant clades. Evolutionary rates of the Pearl River Delta (PRD), YRD-C, and YRD-E clades were independently evaluated under the GTR+G4, strict clock model, and Bayesian SkyGrid tree prior using BEAST (v1.10.4).

To assess the evolution of the variant H7N9 clades, temporal signals were evaluated. This showed that a temporal signal existed (R^2^ = 0.94, Figure S1), indicating that the sampling date could be used for molecular clock calibration in BEAST. The median evolutionary rates (substitutions/site/year) of the RPD lineage (LP) and clades of YRD-C (HP) and YRD-E (LP) estimated by BEAST were 7.74 × 10^−3^ (95% HDP:3.38 × 10^−3^ − 9.08 × 10^−3^), 7.57 × 10^−3^ (95% HDP:5.96 × 10^−3^ − 9.27 × 10^−3^), 5.12 × 10^−3^ (95% HDP:4.27 × 10^−3^ − 5.98 × 10^−3^), respectively (Figure 3). Although the evolutionary rate of the YRD-E clade was slower than that of the others, its population size grew quickly, and it quickly took over in wave 5 (Figure S2).

### Conserved genetic and antigenic variation of the YRD LP H7N9

**Figure 4.**
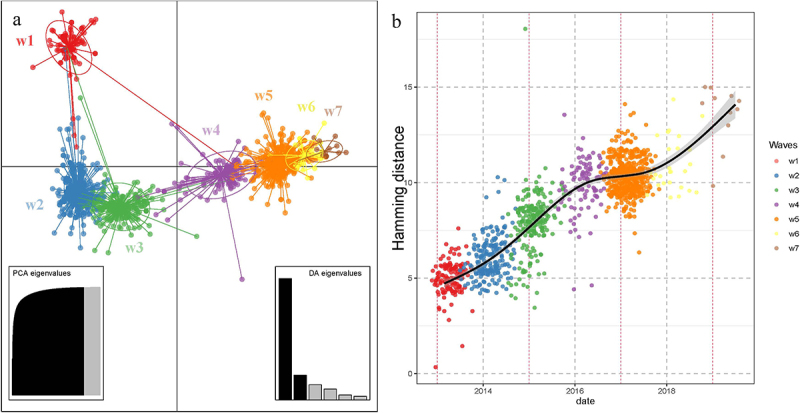
Genetic and antigenic analysis of the YRD lineage. (a) Scatterplots of the DAPC of YRD lineage H7N9. This scatterplot shows the first two principal components of the DAPC of the hemagglutinin gene of H7N9 AIVs using waves of sampling as prior clusters. Waves are shown by different colors and inertia ellipses, whereas dots represent the individual strains. (b) Jitter scatter plot of hamming distance of HA1 antigen sites at different waves of H7N9. The horizontal axis represents time (waves), the vertical axis represents the cumulative hamming distance values, and the scatter colors represent different separation waves. As shown in the legend, the gray areas on both sides of the black line represent the error range of curve fitting.

We used the DAPC and Hamming distance to depict population genetic clusters and antigenic variation, respectively. In the data transformation of the DAPC analysis, we retained the first 124 principal components of PCA, which contained more than 98.3% of the genetic variation. The first two principal components of DAPC can summarize the evolution of the virus population. The first principal component of the DAPC revealed the accumulation of genetic variation from waves 1 to 7 of the H7N9 epidemic (Figure 4a, horizontal axis). Our findings indicated that the seven waves of H7N9 can generally be divided into three large clusters based on the genetic distance of HA, including waves 1, waves 2–3, and waves 4–7. The epidemic strains of Wave 1 were clearly separated from the other clusters in the second principal component (Figure 4a, vertical axis), indicating that more genetic changes accumulated during Wave 1 than during the other epidemic waves. The most likely reason was that genetic changes were driven by adaptation to the new host since the H7N9 AIV jumped to poultry in 2013. Genetic variations usually favor rapid adaptation after host switching to optimize viral fitness [41], which supports the hypothesis that genetic changes during wave 1 are driven by adaptation to a new host. While this may be true for Wave 1, the other clusters also exhibited clear separation in the second principal component. The genetic distances between waves 5 and 4 or 6 were smaller, indicating that less genetic variation accumulated in the viral population during waves 4–6.

The Hamming distance of the antigenic sites was plotted as a scatter and curve to draw insight into the H7N9 antigenic variation. As shown in Figure 4b, our results demonstrate that the Hamming distance of the antigenic sites in the YRD clade constantly increased with time. However, compared with the sharp accumulation rate in waves 1–3 and the following wave 5, the rate in waves 4–5 was flat rather than steep, implying that the antigenicity of the YRD lineage H7N9 drifted slowly over waves 4–5. The line in waves (6 and 7) following wave 5 was steeper due to the national implementation of vaccination, which might cause rapid mutation [15,19]. The antigenicity of the influenza virus is mainly determined by the amino acids on the head of HA (HA1) [42], although the weight of the antigenic sites affecting antigenicity is inconsistent [43]. Our results indicate that the genetic and antigenic variations of the YRD clade in wave 5 were attenuated.

### Few amino acid substitutions on the trunk of YRD-E LP H7N9

**Figure 5.**
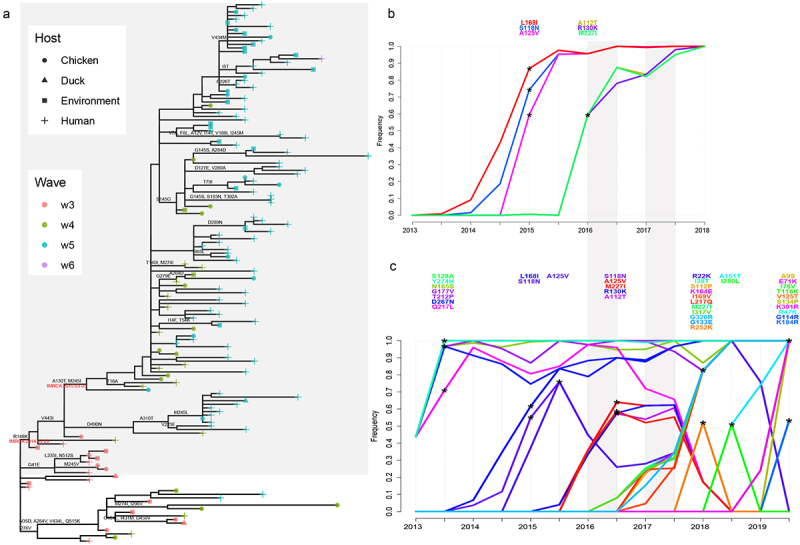
Amino acid adaptation analysis. (a) Amino acid substitutions on the YRD-E clade (light grey background) and the tMRCA of A130T & M245I (March 2015). (b) Sweep Dynamics (SD) plot of the HA protein of the LP H7N9 YRD-lineage dataset (*n* = 320). (c) SD plot of the HA protein of the whole dataset (*n* = 454). The plot shows only significant substitutions. Each line represents the frequency of amino acid mutations, with a significant mutation marked by an asterisk and the mutation site listed above the graph.

Nonsynonymous mutant amino acids along the HA evolutionary tree were explored using the treesub. The amino acid substitution results showed that multiple amino acid site substitutions occurred on the trunk of the tree from 2013 to 2019. Most substitutions were on HA1, whereas only a few substitutions were found on HA2 (Figure. S3). Among these amino acid substitutions, HA1, A143V, G195V, and Q235L (H7 full-length numbering) were in the receptor binding site, A130T, A143V, R148K in the Antigen A site, S136N, G195V in the Antigen B site, and L186I, Q235L in the Antigen D site. The branch of YRD-E exhibited a comb-like topology, which is a typical outbreak branch structure (Figure 5a). The R148K mutation first appeared in this clade in September 2014 (2014.6435), and the A130T and M245I substitutions first emerged in March 2015 (2015.2953). However, few substitutions were found on the trunk of the amino acid substitution tree, indicating that the YRD-E clade had few variations since wave 3.

### LP H7N9 has lacked selective sweep mutations since 2016

Selective sweep mutations have been fully explored to interpret the mutations that may be beneficial to the viral population. The SDplot provides an overview of the specific mutations in the viral genome that are beneficial to population fitness and helps to identify selective sweeps as well as the period during which these mutations provide the virus with a selection advantage [34]. The frequency of each amino acid substitution in the tree was evaluated using the aligned sequences and reconstructed phylogenetic tree. We identified 6 amino acid substitutions that belonged to selective sweep mutations using the YRD lineage dataset (Figure 5b). Since 2014, the frequencies of L168I, S118N, and A125V have significantly increased. In 2016, A112T, R130K, and M227I mutations were found at much higher frequencies. However, insufficient selective sweep mutations were identified in the LP H7N9 YRD lineage from 2016 until they completely disappeared. In contrast, 38 selective sweep mutations were determined using the entire dataset, and 28 were found since 2016 (Figure 5c), suggesting that these mutations were not raised in the LP YRD but in other clades. Our results showed that the YRD lineage has lacked selective sweep mutations since 2016.

### Low epidemic fitness of the LP H7N9

**Figure 6.**
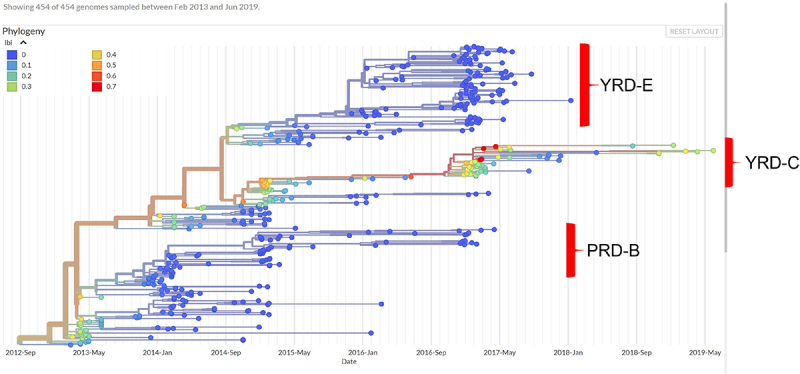
The local branching index (LBI) estimates the epidemic fitness of the H7N9 viruses. The horizontal axis of the evolutionary tree represents time rather than the branch length.

To assess the fitness of H7N9 viruses, we used the LBI as an indicator of individual strain variation. Our results showed that most of the LP H7N9 viruses at shallow nodes (nodes near the tips of the tree, blue tips) had low LBI values (Figure 6). In contrast, only a few LP H7N9 strains exhibited high LBI values in the deep nodes (near the root of the tree). In contrast, the strains in the YRD-C clade have relatively high LBI values. Our results suggest that viruses in the YRD-E clade (LP H7N9) generally have low epidemic fitness. In contrast, viruses in the YRD-C clade (HP H7N9) showed high epidemic fitness.

### Conserved evolution of the YRD-E LP H7N9

**Figure 7.**
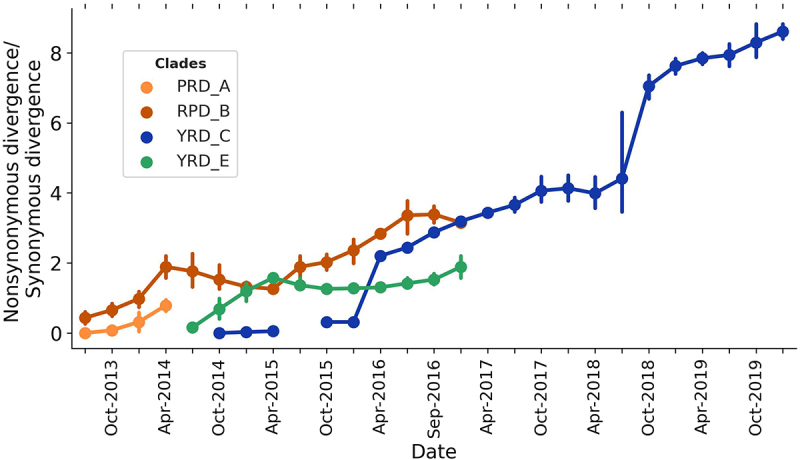
Ratios of accumulation of synonymous and nonsynonymous divergence among the different H7N9 clades.

We adopted the nonsynonymous and synonymous Hamming distance divergence measurements to replace the classical method for calculating the time course of dN/dS ratios. The accumulation of nonsynonymous to synonymous (*dN/dS*) ratios in different clades was estimated using a 6-month time window between 2013 and 2019. The H7N9 viruses essentially diverged from the root as the *dN/dS* ratio increased (Figure 7). The PRD-A clade was short-lived and disappeared after mid-2014. Notably, the divergent ratio of the YRD-E clade first grew to 2, and then unexpectedly remained steady from April 2015 until it disappeared. PRD-B only has a small portion and disappears in wave 5. In comparison, the divergence of the YRD-C (HP H7N9) clade increased. The YRD-C ratio sharply increased following the national H7N9 vaccination campaign conducted in September 2017. The stagnant status of the YRD-E clade may indicate that the viruses in this category had undergone conserved evolution until the time of vanishing.

## Discussion

Our study employed a series of statistical analyses, including principal component analysis (PCA) and discriminant analysis of principal components (DAPC), to assess the conserved genetic and antigenic evolution of LP H7N9. These methods allowed us to identify genetic clusters and track evolutionary patterns. Additionally, we conducted a comprehensive analysis of amino acid substitutions using a sliding window approach to quantify the rate and distribution of changes across the viral genome. These analyses were supported by empirical data from sequence alignments and phylogenetic trees constructed from a dataset of 454 H7N9 isolates collected over five years. The results indicate a low rate of amino acid substitutions, genetic and antigenic conservation, consistent with the observed poor fitness of the LP H7N9 lineage.

Our retrospective investigation showed that LP H7N9 predominated in China’s main poultry-rearing area, which was almost simultaneous nationwide outbreak around the end of 2016 and peaked in wave 5 (Figure 1b and S4). Considering a large number of infections in birds and humans caused by H7N9 AIVs in wave 5 [44,45] and inspiration from sustained mass vaccination strategy that once contributed to the clearance of clade 7.2 H5N1 which once circulated in poultry in northern China [46–48], the development of the bivalent H5 (Re-8) and H7 (Re-1) vaccine in 2017 was a strategic response to the widespread outbreaks of H7N9 in China’s main poultry-rearing areas [9]. This vaccine was designed to target both H5 and H7 subtypes, aiming to provide broad protection against these highly pathogenic avian influenza viruses. This targeted approach has been effective in reducing the incidence of H7N9-associated zoonotic infections in humans and the prevalence of H7N9 in poultry [9,49,50]. The predominance of LP H7N9 before vaccine deployment suggests that the virus had already established a significant presence in the majority area of China, which was subsequently mitigated through vaccination. The success of the bivalent vaccine in clearing LP H7N9 highlights the importance of timely and targeted vaccination strategies in controlling avian influenza outbreaks.

In this study, we applied multiple comprehensive bioinformatics methods and found that the conserved genetic and antigenic evolution of LP H7N9 led to insufficient amino acid substitutions and low fitness. Because of its evolutionary conservation, it is postulated that the YRD-E clade of LP H7N9 May be more susceptible to extermination through extensive vaccination, particularly in a shortened timeframe, attributing it to a lower possibility of antigenic variation, which could potentially influence vaccine efficiency. While the observed amino acid substitutions in the YRD-E LP H7N9 lineage are few, they are nonetheless significant. Through comparative analysis with known functional domains and antigenic sites of H7N9 viruses, we don’t have identified potential implications for viral fitness and antigenic properties. These substitutions may contribute to the virus’s evolutionary trajectory by influencing its ability to evade the host immune response and maintain low pathogenicity. Further experimental validation is required to fully elucidate the functional and antigenic consequences of these genetic alterations.

Amino acid substitutions are changes in genetic codons that alter the protein structure and function of the virus. Some of these changes may be beneficial, neutral, or deleterious to the viruses. Beneficial changes may increase viral fitness, while deleterious changes may decrease it. Neutral changes may have no effect or a very small effect on viral fitness. However, insufficient amino acid substitutions during the evolutionary trajectory could mean that the virus is unable to adapt to changing environmental conditions or host immune responses [51]. Fitness declines in the absence of adaptive mutations due to a changing environment or the accumulation of deleterious mutations [35]. This could reduce the fitness of a viral population, making it less likely to survive and spread. Therefore, rapidly evolving viruses typically employ various strategies (e.g. mutation, recombination, or reassortment) to increase their genomic diversity presumably to improve fitness and/or evade host immune responses [52]. As a result, the genomic frequency of viral mutations that enhance epidemiological fitness increases [53].

The YRD-E clade of viruses has evolved conservatively, notably lacking in trunk mutations and selective sweep mutations when compared to other H7N9 clades, particularly the highly pathogenic (HP) H7N9 variants. This absence of key mutations is a distinguishing genetic trait of the LP H7N9 lineage. To better understand this characteristic, we conducted a comparative genetic analysis across various H7N9 clades, including the HP H7N9 strains. Our analysis revealed that HP H7N9 clades are marked by a higher incidence of selective sweep mutations and trunk mutations, indicative of a more active evolutionary environment. Conversely, LP H7N9 maintains a genetic profile characterized by stability and minimal variation. This comparative study emphasizes the distinct evolutionary path followed by LP H7N9, suggesting that the absence of these mutations could be associated with its reduced fitness and limited adaptability to changing environmental conditions or host immune responses. The sustained lack of selective sweep mutations since 2016 in the LP H7N9 lineage indicates a period of genetic stability and the absence of significant positive selection. This stability might be a reflection of the virus’s adaptation to a specific ecological niche or a decrease in evolutionary pressure due to effective control measures, such as vaccination [54]. The infrequent occurrence of recent selective sweeps also suggests a diminished potential for antigenic shifts or substantial functional changes that could affect pathogenicity [55].

DAPC and HMD analyses of the YRD lineage LP H7N9 viruses have also indicated attenuated genetic and antigenicity evolution since 2015. Insufficient synonymous and nonsynonymous mutations in LP H7N9 viruses during the evolutionary trajectory could potentially diminish viral population fitness to survive and impair the beneficial effects of mutations that contribute to adaptive evolution. Thus, the evolutionary conservation of LP H7N9 could reduce viral diversity and respond to their low fitness, as detected by LBI. Ding et al. also found that the genotypic diversity of H7N9 was significantly reduced in waves 4–5 [56]. Conversely, HP H7N9 (YRD-C clade) has high fitness values and has a shaped increase in accumulating *dN/dS* ratios, which may explain why the HP H7N9 virus is still circulating in northern China and evolves rapidly under vaccination [14,15,19]. In this study, we found that the predominant outbreak of LP H7N9 AIVs has undergone conserved evolution since 2016, resulting in this clade being unable to acquire sufficient adaptive mutations in wave 5, thereby reducing the fitness of the viral population. We conclude that this conserved evolution could accelerate viral elimination or eradication during vaccination.

Several factors have been hypothesized to explain the disappearance of a certain subtype of the virus among host populations. First, a cohort study showed that SARS-CoV-2 displaced other seasonal respiratory viruses [57], The emerging Omicron variant has displaced the global prevalence of Delta SARS-CoV-2 over a short span [58]. Though the HP H7N9 AIVs were barely isolated in the Yangtze River Delta area, the LP H7N9 AIVs also disappeared in this area (https://nextstrain.org/flu/avian/h7n9/ha?f_division=Anhui,Jiangsu,Shanghai,Zhejiang). Therefore, it is unlikely that the HP strains were replaced by LP H7N9. Second, Palese and Wang proposed that old seasonal influenza virus strains are cleared by a population-level humoral immune response triggered by pandemic strains against conserved hemagglutinin stalk epitopes and/or neuraminidase proteins [59]. Examples include the seasonal H1N1 and 2009 H1N1 pandemics. H7Ny and H×N9are not prevalent in H7N9 in China. Thus, the LP H7N9 AIV would not be diminished by humoral immunity elicited by the other subtypes.

Third, Yan et al. proposed that the immune footprint of ancestral strains promotes competition between related emerging viral variants via cross-reactivity and can drive all but one competitive lineage of rapidly adapting pathogens [60]. The host population exerts selection pressure on the viral population, causing the viral population to evolve toward more distant areas of antigenic space from the host population [61]. Antigenic escape studies using polyclonal human sera have shown that successful viruses must acquire multiple beneficial mutations with significant effects to successfully escape the diversity of host immunity [62]. Therefore, sufficient mutations need to be accumulated for progeny viruses to escape the cross-immunity of ancestral strains. Otherwise, the viral population would attenuate or die out. For instance, the Zika virus spread widely across Latin America between 2015 and 2017, resulting in extensive population immunity (approximately 50%). However, the viral population does not evolve antigenically to avoid herd immunity, which limits its capacity for persistent viral transmission [60,62,63]. Another example is that Yamagata lineage influenza B viruses have been rarely detected and have disappeared globally since 2020 [64–66]. Although viruses of the Yamagata lineage are somewhat diverse at the genomic level, amino acid substitutions appearing in the HA protein have not caused significant changes in their antigenicity since 2015. Thus, the conserved antigenic variation may be one of the reasons that caused the “extinction” of Yamagata lineage viruses in the population [64]. Conversely, the rapid adaptive mutations in the S1 protein of SARS-CoVA-2 drive clade success, which was also significantly correlated with the clade growth rate [67].

Regarding LP H7N9, we also observed the conserved genetic and antigenic evolution of HA before vaccine usage (September 2017), which might have undermined its fitness. The conserved evolution of the LP H7N9 AIVs is characterized by minimal genetic and antigenic variation, which may have little impact on viral fitness and pathogenicity and may instead have the potential to improve vaccine efficacy. Instead, the sustained circulating HP H7N9 AIV in North China under the same vaccination procedure might be attributed to its rapidly increasing *dN/dS* ratios since it emerged in the middle of 2016, which was beneficial to its high fitness. Previous studies have shown that H7N9 AIVs have accelerated their evolution under vaccination pressures [14,15,19]. Therefore, the rapid increase in the *dN/dS* ratio of the HP H7N9 YRD-C clade was most likely due to rapid nonsynonymous mutations under natural selection pressure and vaccination. Therefore, we assume that the conserved evolution of LP H7N9 attenuates the virus population size and accelerates virus elimination during vaccination. The maintenance of this conserved evolutionary state of the virus may be related to selective restrictions that maintain functional adaptations of the virus (e.g. receptor binding) [68].

The low epidemic fitness of the LP H7N9 lineage, as indicated by the LBI values, suggests a reduced capacity for rapid spread and adaptation. This observation has significant implications for pathogenicity, as lower epidemic fitness may correlate with reduced virulence. Additionally, the stability of the LP H7N9 lineage may enhance vaccine efficacy by limiting the emergence of antigenic variants that could escape vaccine-induced immunity. Furthermore, the reduced transmission potential of LP H7N9 could contribute to lower epidemic prevalence, thereby mitigating the impact of the virus on public health.

The Local Branching Index (LBI) serves as a practical metric for estimating viral fitness via phylogenetic patterns, yet its limitations are significant. LBI values are contingent on the phylogenetic tree’s structure and may not comprehensively reflect viral fitness complexity across all situations. Derived from the density of branching events near a sequence, LBI can be impacted by sampling bias and non-uniform sequence distribution. Consequently, while LBI is a useful indicator of relative fitness within a phylogenetic context, its interpretation demands careful consideration and should be corroborated with additional fitness metrics and empirical data. LBI’s limitations are rooted in its phylogenetic tree-based calculation. High-fitness viruses typically display dense branching, whereas low-fitness viruses lack such features [36]. This reliance on tree structure renders LBI susceptible to inaccuracies from inadequate or uneven sampling. The influence of sample size on LBI-based fitness estimates requires further scrutiny to ensure their accuracy.

The absence of descendant branches in the YRD-E clade could contribute to low LBI values, but these values do not definitively signify low fitness. The paradox of widespread YRD-E clade viruses with low LBI in wave 5 suggests multifaceted evolutionary dynamics, warranting further investigation. Seasonality likely influenced H7N9’s decline in Period 4 (summer 2017), aligning with influenza characteristics [1,69]. Our prior study revealed a viral population reduction in the summer of 2017 [19], and a sharp drop in LP and HP H7N9 isolations by mid-2017, as shown by Nextstrain data (https://nextstrain.org/flu/avian/h7n9/ha). Additionally, H7N9’s limited host range, primarily affecting poultry, may enhance its elimination via targeted vaccination.

According to another research findings, the hemagglutinin (HA) gene of the H7N9 virus (A/camel/Inner Mongolia/XL/2020) isolated from camels, exhibited a high degree of nucleotide sequence similarity to that of A/chicken/Shandong/SD69/2015 (H7N9), with 99.82% nucleotide homology [70]. Moreover, this virus displayed substantial genetic relatedness to H7N9 strains previously identified in humans and environmental samples in Inner Mongolia in 2017, with nucleotide homology ranging from 87.8% to 97.8%. This evidence suggests that the H7N9 virus isolated from camels in Inner Mongolia in 2020 has undergone minimal molecular changes compared to the viruses from 2017, indicating a potential conservation in the evolutionary trajectory of the LP H7N9 virus. The recurring detection of LP H7N9 in camels also raises concerns about the zoonotic potential of this virus, emphasizing the need for ongoing surveillance and research to understand its epidemiology and to assess the risk it poses to public health.

Our study indicates a strong correlation between the execution of mass vaccination campaigns and the subsequent decline in LP H7N9 prevalence. However, it is crucial to recognize the inherent limitations of our research. While our analysis sheds light on the evolutionary patterns of LP H7N9 and the possible influence of conserved evolution on viral fitness and vaccine effectiveness, these insights are grounded in observational data and theoretical frameworks. It is important to note that the observed correlation does not definitively prove causality. Various factors, such as alterations in surveillance practices, environmental shifts, and other concurrent disease management strategies, could also significantly impact the observed trends. To unravel the causal links and the multifaceted factors that underpin the efficacy of vaccination against H7N9, it is imperative to conduct further studies using experimental designs, longitudinal surveillance, and detailed data analysis.

In summarizing the outcomes of our investigation, it is imperative to reiterate that this study is fundamentally observational. Our analysis revealed a significant association between the implementation of mass vaccination campaigns and a subsequent reduction in the prevalence of the low pathogenic (LP) H7N9 avian influenza virus. However, it is critical to acknowledge that these findings do not establish causality but suggest a potential linkage that warrants further exploration. Several factors, including but not limited to changes in surveillance efforts, environmental changes, and other concurrent disease control measures, may also play significant roles in the observed trends. Further studies employing experimental designs, longitudinal monitoring, and more granular data analysis are essential to elucidate the causal pathways and factors contributing to the effectiveness of vaccination strategies against H7N9.

## Supplementary Material

Supplemental Material

## Data Availability

Data were derived from the public database and 10.1016/j.meegid.2021.104993.

## Highlights

1. Mass vaccination is associated with a significant reduction in the detection of LP H7N9 in China, whereas HP H7N9 has persisted because of its ability to escape vaccination.
2. The YRD-E clade of LP H7N9 showed a tardy evolutionary trajectory, characterized by the conservation of its genetic and antigenic variation, minimal amino acid substitutions along its phylogenetic tree, and lower epidemic fitness than the HP variants.
3. The YRD-E clade of LP H7N9 is hypothesized to be more susceptible to elimination through mass vaccination in a relatively short period owing to its lower probability of antigenic variation that might affect vaccine efficiency.

## Importance

Mass poultry vaccination has effectively decreased low pathogenic (LP) H7N9 in China; however, highly pathogenic (HP) H7N9 has persisted because it escapes vaccination. This study showed that the elimination of LP H7N9 is due to its slow evolution. HP strains continue to circulate because they change in ways that help them avoid being blocked by the vaccine. The researchers closely examined the genetic evolution of these viruses and found that the LP viruses changed little from 2016 until they disappeared. However, HP viruses evolve rapidly and mutate to evade vaccines. In conclusion, analyses of H7N9 avian influenza viruses in China revealed significant differences between the evolutionary trajectories of LP and HP H7N9 viruses under vaccination, with implications for control strategies. Continuous vaccine updates and close monitoring of avian influenza viruses are key to eradicating H7N9, which is a threat to both birds and humans.
