# Supplementary figures and images for: Characterization of Conserved Evolution in H7N9 Avian Influenza Virus Prior Mass Vaccination
Source: Virulence. 2024 Sep 6;15(1):2395837. doi: 10.1080/21505594.2024.2395837 (PMC11382709; doi:10.1080/21505594.2024.2395837)

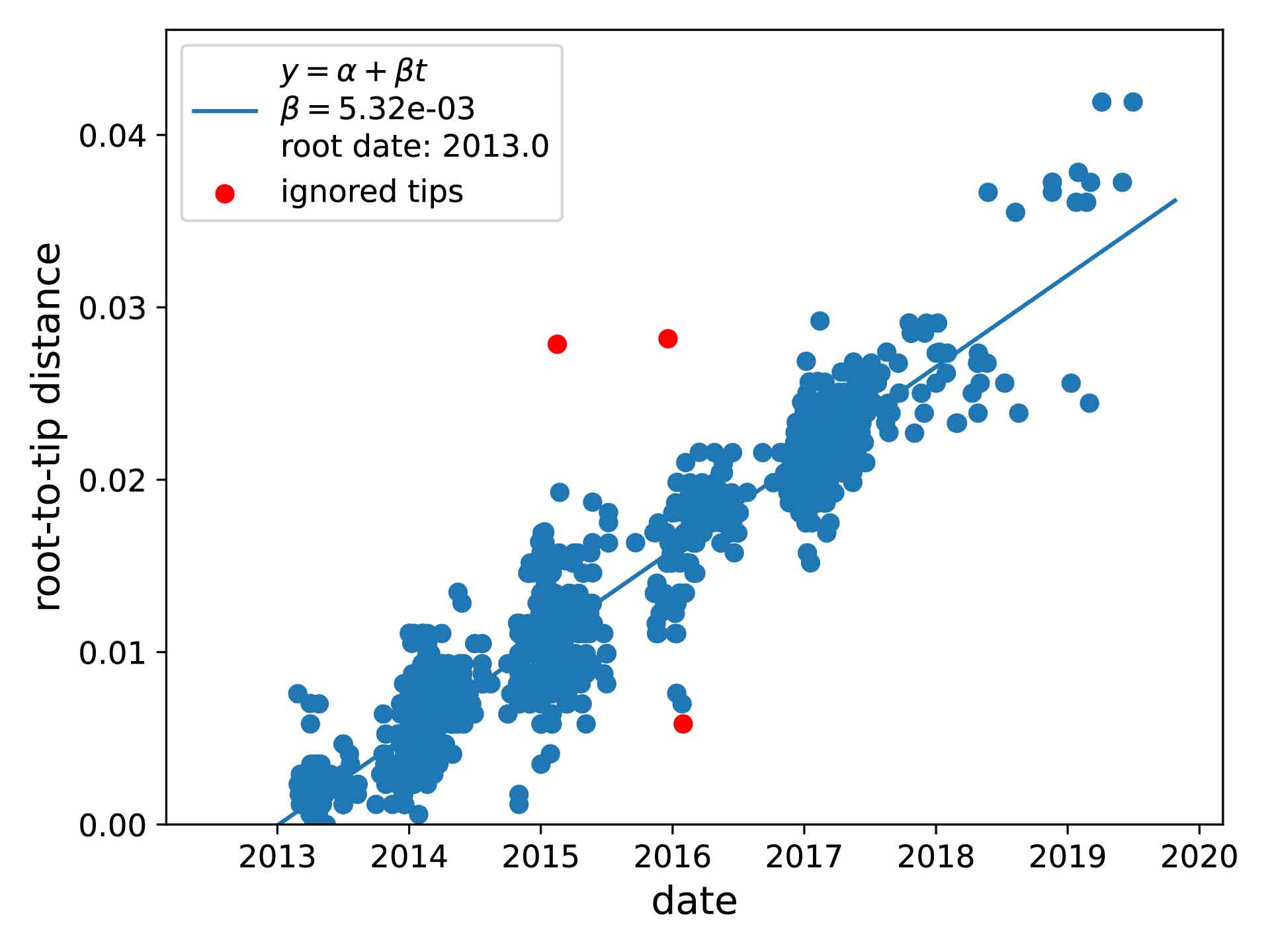

Supplement: Supplemental Material [file KVIR_A_2395837_SM5793.zip › Figure_S1_root_to_tip_regression.jpg]

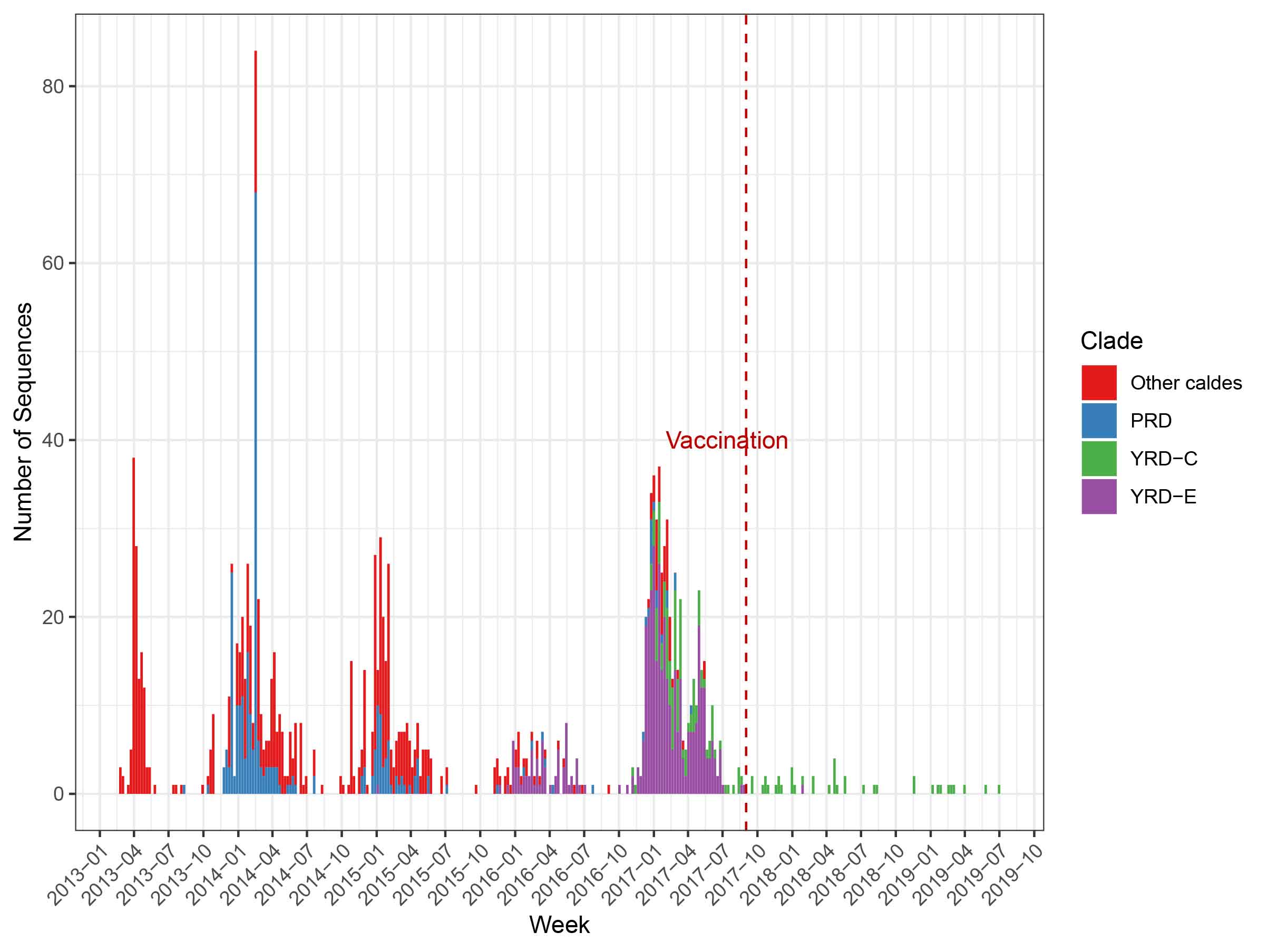

Supplement: Supplemental Material [file KVIR_A_2395837_SM5793.zip › Figure_S2_H7N9_clade_against_week.jpg]

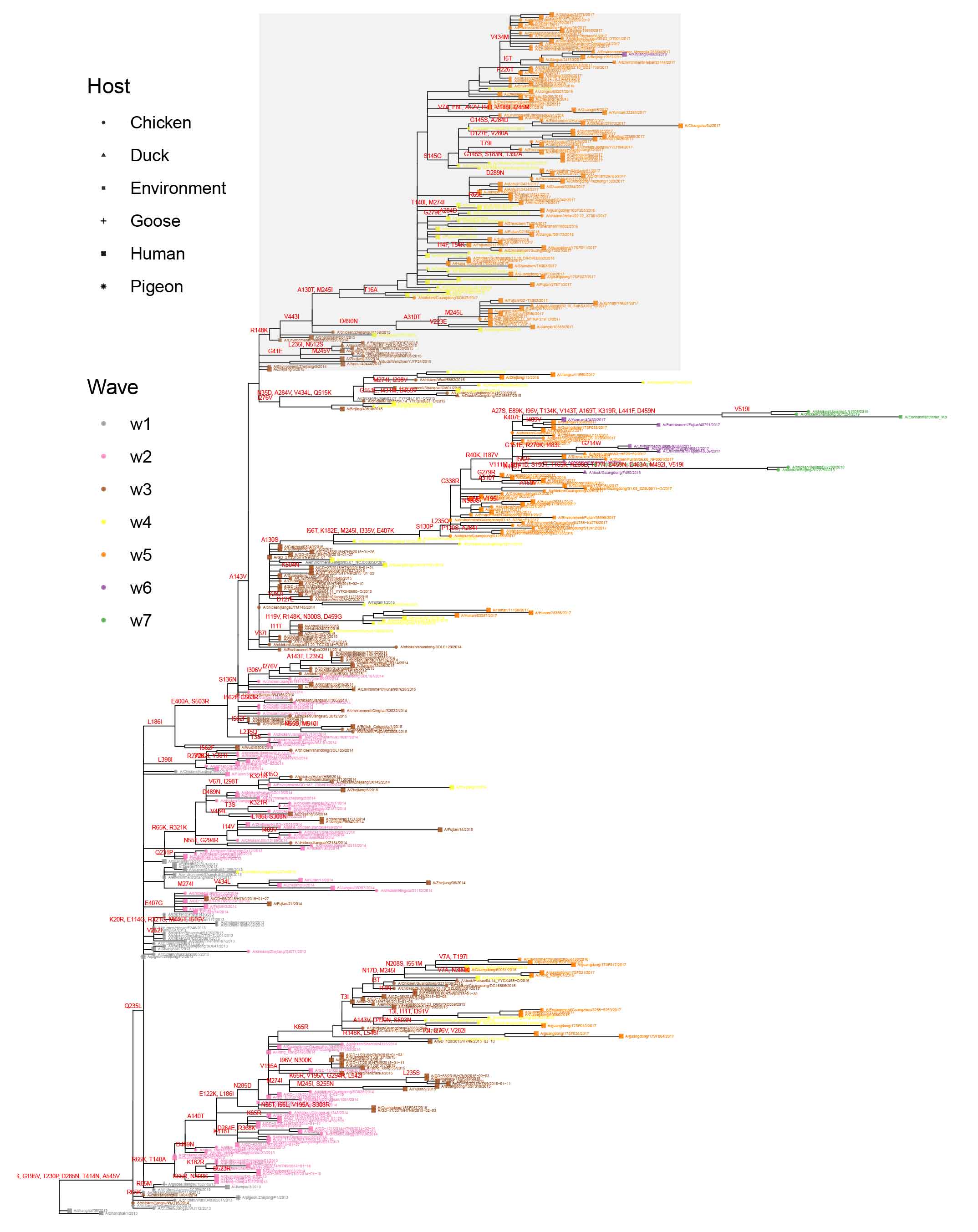

Supplement: Supplemental Material [file KVIR_A_2395837_SM5793.zip › Figure_S3_H7N9_HA_454_treesub.jpg]

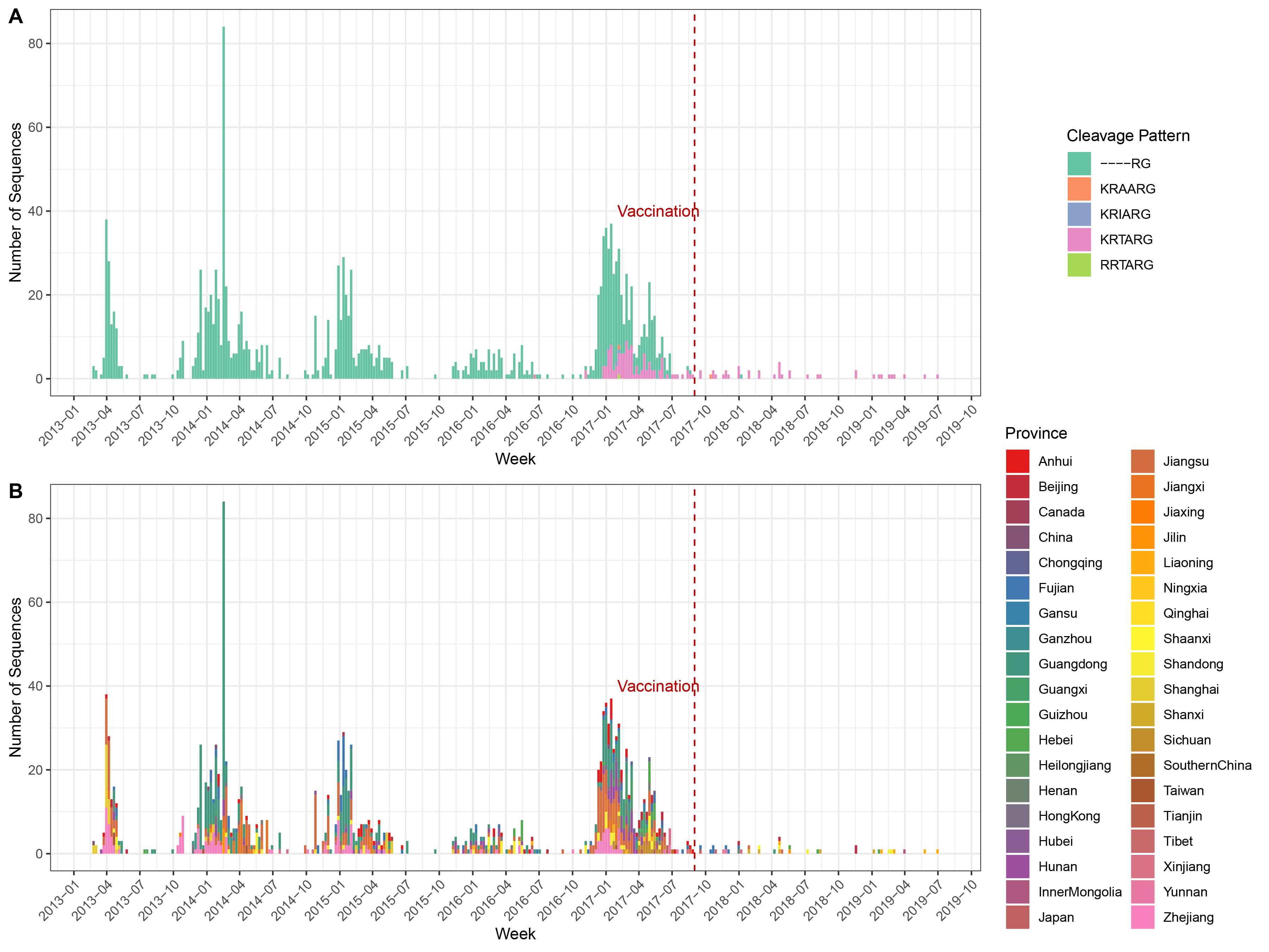

Supplement: Supplemental Material [file KVIR_A_2395837_SM5793.zip › Figure_S4_H7N9_cleavage_Province_against_week.jpg]
